# Subcellular Localization of CD24 Is Associated with Integrin β1 Signaling, Immune Suppression, and Clinical Outcomes Across Human Cancers

**DOI:** 10.3390/biology15181611

**Published:** 2026-09-12

**Authors:** Bhaumik Patel, Marina Curcic, Mohamed A. Eltokhy, Faizah Alabi, Omar Eltoukhy

**Affiliations:** 1 Department of Immunotherapeutic and Biotechnology, Texas Tech University Health Science Center, Abilene, TX 79601, USA; mcurcic@ttuhsc.edu (M.C.); mohamed.eltokhy@ttuhsc.edu (M.A.E.); faialabi@ttuhsc.edu (F.A.); 2 Department of Clinical Pharmacy, Misr International University, Cairo 11566, Egypt; omar.ashraf@miuegypt.edu.eg

**Keywords:** CD24, Siglec-10, integrin β1, SRC, MEK1, TGFβ1, macrophage, T and NK cells, overall survival

## Abstract

CD24 is an emerging innate immune checkpoint that regulates inflammatory and antiviral immune responses through interactions with Siglec family receptors. Although CD24 has been extensively studied in immune regulation and has recently gained attention as a therapeutic target in infectious and inflammatory diseases, its role in cancer remains incompletely understood. CD24 is overexpressed in multiple human malignancies, where accumulating evidence suggests that it promotes tumor progression and immune evasion through activation of diverse intracellular and extracellular signaling pathways. However, the importance of its subcellular localization and associated signaling remains poorly defined. Here, we investigated CD24 expression, localization, signaling associations, immune-cell infiltration, and clinical outcomes using publicly available cancer datasets, focusing on breast cancer (BRCA), cervical squamous cell carcinoma (CESC), and glioblastoma multiforme (GBM). Our analyses revealed prominent membrane-associated CD24 localization in BRCA and CESC, whereas CD24 was predominantly cytoplasmic in GBM. BRCA and CESC exhibited increased integrin β1 expression and distinct associations between CD24 and components of integrin β1/SRC and TGFβ-associated signaling networks, including MEK1 and SMAD7. In addition, we identified a structurally consistent and energetically favorable predicted interface between CD24 and Siglec-10, suggesting a mechanism by which CD24 may suppress anti-tumor immunity. Elevated CD24 expression was associated with immune-infiltration patterns consistent with an immunosuppressive tumor microenvironment in BRCA and CESC, including negative associations with M1 macrophage, CD8^+^ T-cell, and NK-cell infiltration. Importantly, high CD24 expression was associated with unfavorable overall survival in BRCA and CESC, whereas the opposite relationship was observed in GBM. Collectively, these findings suggest that the subcellular distribution of CD24 may be associated with distinct tumor signaling, the immune microenvironment, and prognostic profiles, highlighting the potential importance of CD24 localization in determining its tumor-type-specific biological functions.

## Simple Summary

CD24 protein plays an important role in the regulation of immune escape signaling. Overexpression of *CD24* mRNA and protein has been observed in multiple cancers such as breast cancer, cervical cancer and glioblastoma multiforme. Membrane-localized CD24 protein facilitates integrin β1/SRC/TGFβ-associated signaling networks in breast and cervical cancer. Moreover, CD24-Siglec-10 interaction mediates a negative association of M1 macrophages, CD8^+^ T-cells, and NK cells, leading to an immune-suppressive tumor microenvironment, which predicts unfavorable overall survival in breast and cervical cancers. Thus, membrane localization of CD24 is crucial for the activation of intracellular signaling, immune escape and predicting clinical outcomes in cancer.

## 1. Introduction

One of the highly glycosylated cell-surface proteins responsible for immune homeostasis, cellular communication, and even cellular differentiation is the Cluster of Differentiation 24 (CD24). The presence of CD24 was initially linked to B-cell differentiation. Since then, the presence of CD24 has been linked to various physiological as well as pathological signaling networks [1]. The structure of mature CD24 molecules is essential to its function. It consists of a short extracellular peptide backbone anchored to the cell membrane via glycosylphosphatidylinositol (GPI). The presence of the dense glycan coating is essential for the recognition of CD24 in its targets, making it essential for its activity [2].

CD24 regulates several physiological processes. For instance, it regulates the innate immune response and maintains tissue homeostasis. This is achieved by CD24 interaction with Sialic acid-binding Ig-like lectin 10 (Siglec-10). The activation of Siglec-10 results in an inhibitory response towards any excessive inflammatory response that might arise [3]. Moreover, CD24 was found to support other physiological processes such as cell adhesion, cell apoptosis, and tissue remodeling. Several emerging pieces of evidence suggest that CD24 does not act only as a direct membrane receptor; its role goes even further, functioning as a membrane-associated molecular fine-tuning receptor that modulates signaling intensity. This might explain the effect of CD24 dysregulation in multiple pathological conditions, such as malignancies and autoimmune diseases [4,5].

The role of CD24 in malignancies was further investigated after it was confirmed to be one of cancer stem cell markers. The expression of CD24 was directly linked to the enhancement of tumorigenic potential, tumor spreading, and adaptation to hostile microenvironments [2]. The association between CD24 expression and cancer stem cells highlights its potential as a therapeutic target to disrupt tumor initiation and progression at their cellular origin. This effect is evident via the suppression of CD24 expression, which resulted in impaired tumor initiation, decreased metastatic potential, and increased therapeutic sensitivity in several cancer models. Thus, targeting CD24 expression might be a potential target for several resistant tumors [6].

The role of CD24 in tumors is not exclusive to its recognized use as a stem cell marker. The presence and expression of CD24 is now related to tumor aggressiveness. The increased expression of CD24 was correlated with advanced-stage tumors in multiple cancers, such as breast, cervical, prostate, bladder, and lung cancers [1]. The suggested mechanism of action behind this effect is the modulation of several pathways related to cancer survival. Different preclinical studies have demonstrated the effect of CD24 expression on activation of proliferation pathways such as PI3K/AKT, MAPK/ERK, STAT3, Wnt/β-catenin, Hedgehog, and NF-κB [7]. Furthermore, CD24 was found to be directly related to tumor escape by acting as an immune checkpoint through its interaction with Siglec-10 expressed on immune cells. The CD24–Siglec-10 pathway normally maintains tissue homeostasis by limiting excessive inflammation following tissue damage. In cancer, however, tumor cells hijack this pathway to avoid immune recognition and escape macrophage-mediated immune surveillance [8]. This mechanism of immune evasion is a hallmark of cancer and is an important determinant of tumor progression and clinical outcome. Although CD24 predominantly aids in immune evasion, a recent study in triple metastatic breast cancer reported that CD24 promoted fatty-acid β-oxidation and thereby functioned as a potential tumor suppressor in this specific context [9]. Additionally, CD24 was shown to promote proliferation and inhibit apoptosis of cervical cancer cells through the activation of the MAPK pathway [10]. These findings highlight the context-dependent nature of CD24 and support the urge to evaluate its expression and functional significance within individual cancer types rather than considering CD24 a universal oncogenic driver.

Despite increasing recognition of CD24 as an emerging innate immune checkpoint and its documented overexpression across multiple malignancies, the molecular mechanisms underlying its role in tumor progression and immune modulation are not completely understood. In this study, we investigated the mechanistic role of CD24 in cancer biology using publicly available transcriptomic and clinical datasets from The Cancer Genome Atlas (TCGA). We evaluated associations between CD24 expression and subcellular localization and components of integrin β1/SRC and TGFβ signaling networks, together with immune-cell infiltration and clinical outcomes across selected CD24-high cancers. We further performed protein–protein docking to characterize the predicted structural interface between CD24 and Siglec-10. 

## 2. Materials and Methods

ShinyGO V0.741: ShinyGO 0.741 is a specific version of the intuitive, graphical web application used for Gene Ontology (GO) enrichment analysis and pathway visualization. We have used ShinyGO v0.741 (http://bioinformatics.sdstate.edu/go74/, accessed on 9 September 2025) to assess gene ontology enrichment analysis of *CD24*. The ShinyGO biological process chart for the *CD24* gene provides fold-enrichment values for pathways associated with this gene. Fold enrichment is defined as the percentage of genes in the list belonging to a pathway, divided by the corresponding percentage in the background. Fold enrichment indicates the extent to which genes associated with a particular pathway are overrepresented.

STRING: The STRING database demonstrates known and predicted protein–protein interactions, which include direct and indirect associations from computational predictions and are aggregated from other databases. Moreover, STRING also provides functional enrichments, including biological process, molecular function, cellular component, and KEGG pathways. STRING was used to analyze the CD24 protein–protein interaction network and to perform Gene Ontology enrichment analysis of the corresponding *CD24* gene-associated network (https://string-db.org/, accessed on 9 September 2025).

GEPIA2 database: Gene Expression Profiling Interactive Analysis (GEPIA2) serves as an advanced web server for extensive gene-expression-profile, interaction and correlation analysis from TCGA and GTEx databases (http://gepia2.cancer-pku.cn/#index, accessed on 10 October 2025 through 25 August 2026). *CD24* mRNA expression and its correlation with multiple genes were studied using GEPIA2. mRNA data were produced here by downloading recomputed RNA-Seq expression profiles from The Cancer Genome Atlas (TCGA, https://www.cancer.gov/ccg/research/genome-sequencing/tcga, accessed on 9 September 2025) and the Genotype tissue expression (GTEx, https://gtexportal.org/home/). Survival maps and curves by Mantel–Cox and by Kaplan–Meier of *CD24* were studied through the GEPIA2 database.

Human Protein Atlas (HPA): The Human Protein Atlas provides details about all human proteins across all major tissues, organs, cell lines and cancers (https://www.proteinatlas.org/, accessed on 12 September 2025 through 25 August 2026). Expression of multiple proteins such as CD24, MEK1, integrin β1, SMAD4, and SMAD7 were checked using HPA. Immunohistochemistry images of CD24 and MEK1 were used from HPA. According to the published HPA procedure, 1 mm tissue microarray cores were analyzed by conventional brightfield immunohistochemistry using DAB-based detection and hematoxylin counterstaining. Normal and cancer tissues were evaluated using the same standardized HPA annotation criteria, although the specimens were independent and not patient-matched. Quantification of CD24 and MEK1 expressions was performed using ImageJ 1.54g analytical software.

TIMER 2.0: Tumor Immune Estimation Resource 2.0 (TIMER 2.0) is a comprehensive web-based resource for immune infiltration analysis (https://compbio.cn/timer2/, accessed on 6 October 2025, through 25 August 2026). Correlation between CD24 and tumor-infiltrating immune cells was assessed using TIMER 2.0. 

ClusPro: Protein–protein docking was performed using the ClusPro 2.0 web server to predict the interaction interface between human CD24 and Siglec-10. ClusPro is an automated protein–protein docking platform that has reliably demonstrated high forecast accuracy in the Critical Assessment of Predicted Interactions (CAPRI) experiments using physics-based docking and cluster-based ranking. The three-dimensional structures of human CD24 (UniProt: P25063) and Siglec-10 (UniProt: Q96LC7) were obtained from the AlphaFold Protein Structure Database (RRID:SCR_023662; https://alphafold.ebi.ac.uk, accessed on 9 September 2025) in a Protein Data Bank (.pdb) format. The CD24 structure was downloaded on 31 October 2025, and the Siglec-10 structure was downloaded on 3 December 2025.

For docking simulations, CD24 was specified as the receptor and Siglec-10 as the ligand. The ClusPro server performed four independent docking calculations: Balanced, Electrostatic-favored, Hydrophobic-favored, and Van der Waals plus Electrostatics (VdW + Elec). These scoring calculations assign different relative weights to electrostatic, hydrophobic, and van der Waals interactions, allowing evaluation of the predicted protein–protein interface under different physicochemical conditions.

For each scoring mode, ClusPro generated thousands of docking conformations that were automatically clustered according to structural similarity. The resulting clusters were ranked based on cluster size and weighted energy score. Larger clusters with more negative energy values indicated higher docking confidence. To account for variability among individual docking solutions and identify reproducible interaction sites, an ensemble of 24 representative CD24–Siglec-10 docked complexes derived from the top-ranked clusters across the four scoring modes was analyzed. For each complex, residues at the CD24-Siglec-10 interface were identified using a heavy-atom distance cutoff of <5 Å. CD24 and Siglec-10 contact residues were then compared across all 24 complexes to determine the frequency and reproducibility of individual residue contacts. Residues repeatedly identified across the docking ensemble and represented across the different scoring conditions were considered consensus interface residues, whereas residues showing the greatest recurrence across the ensemble were considered core interaction hotspots. This ensemble-based approach was used to identify interaction sites that were robust to differences in the weighting of electrostatic, hydrophobic, and van der Waals contributions.

Structural Visualization: Predicted CD24-Siglec-10 docking complexes generated by ClusPro were visualized and analyzed using the open-source molecular graphics software PyMOL V3.1.0 (RRID:SCR_000305; https://pymol.org). The representative docking models corresponding to the highest-ranked cluster from each ClusPro scoring mode were imported into PyMOL for structural analysis of the predicted interaction interface. For visualization, the individual structures of CD24 and Siglec-10 were displayed using distinct surface colors, and the predicted CD24-Siglec-10 complex was subsequently visualized to illustrate the docking orientation and interaction interface, with key interacting residues identified and labeled. Representative images of the individual protein structures, the docked complex, and enlarged views of the interaction interface were generated following optimization of molecular orientation, residue labeling, and rendering parameters in PyMOL.

Data availability: The data generated in this study were obtained from the Cancer Genome Atlas (https://www.cancer.gov/ccg/research/genome-sequencing/tcga) and the Genotype tissue expression (https://gtexportal.org/home/).

Statistical significance: Data obtained from various tools were analyzed using multiple statistical methods. GEPIA 2 expression analysis data were analyzed by one-way ANOVA (F-test) and survival analysis data were analyzed using the Mantel–Cox test and Kaplan–Meier test. TIMER 2 immune cell infiltration data is computed using the Wilcoxon test. IHC protein expression quantification analysis was presented using GraphPad Prism 11 and statistics were performed using an unpaired *t*-test. *p* values < 0.05 were considered statistically significant. 

## 3. Results

### 3.1. Interaction and Involvement of CD24 in Cellular Pathways

**Figure 1:**
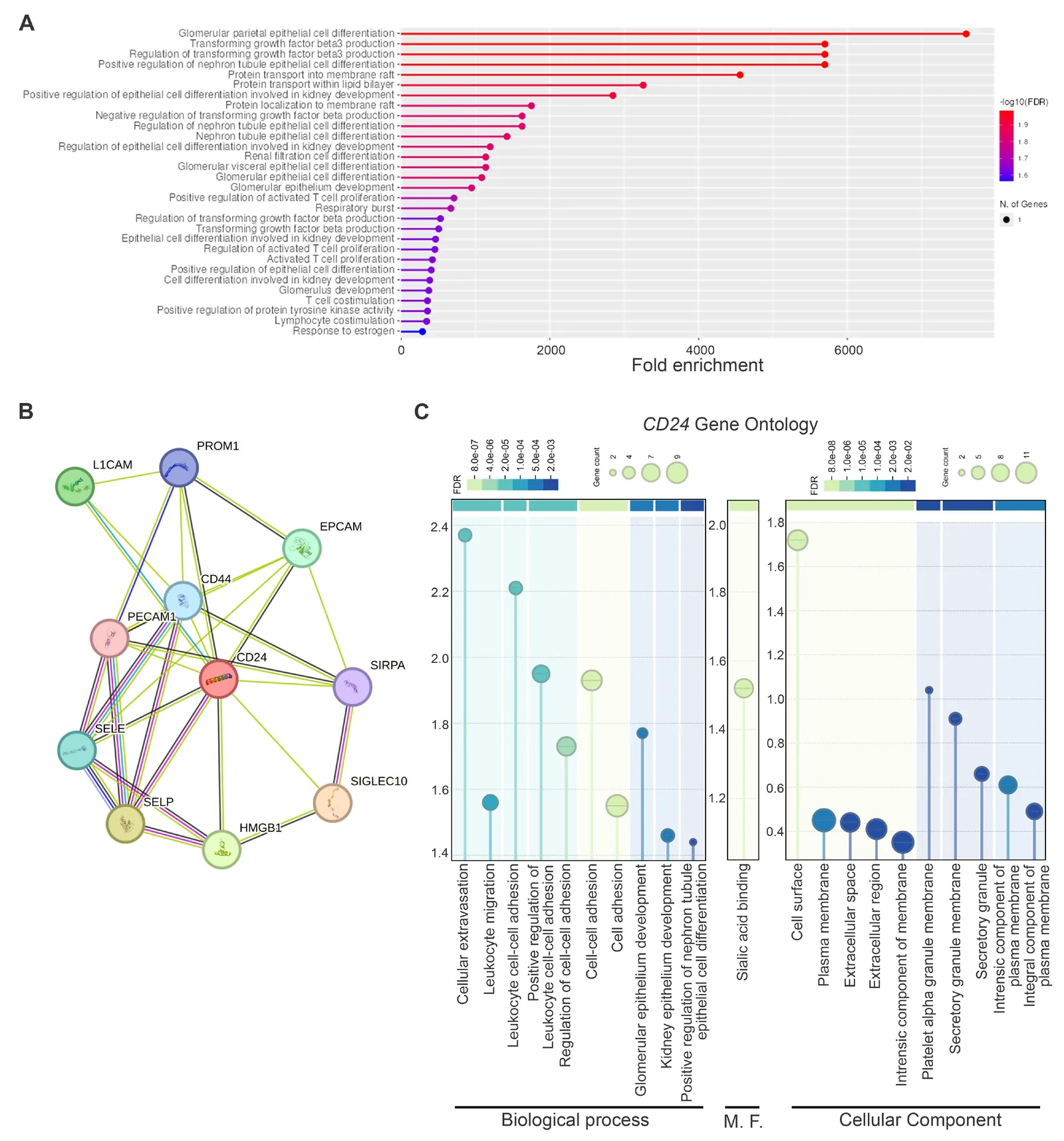
Biological pathway, protein interactions and gene ontology of *CD24*. (**A**) Gene Ontology enrichment analysis of *CD24*, showing its involvement in multiple pathways. (**B**) Direct and indirect interaction of CD24 with associated proteins. (**C**) Gene Ontology enrichment analysis of *CD24* presenting three aspects: biological process, molecular function and cellular component.

CD24 is expressed heavily in hematopoietic cells such as B-cells, developing T-cells, neutrophils, and macrophages. Moreover, expressions are also present in non-hematopoietic cells such as neural cells, keratinocytes as well as progenitor and stem cells. We performed the ShinyGO v0.741 gene ontology enrichment analysis of *CD24*, which indicated the involvement of *CD24* in multiple pathways, such as regulation of TGFβ3 production, protein transport within lipid bilayer, and activator of protein tyrosine kinase. *CD24*-associated immune functions include interactions with macrophage, positive regulation of activated T-cell proliferation, and localization to the external side of the plasma membrane (Figure 1A). Furthermore, the STRING analysis suggested that CD24 interacts with epithelial cell-associated proteins EPCAM, L1CAM and PECAM1, stem cell-associated marker CD44 and macrophage-related protein Siglec-10 (Figure 1B). An Additional Gene Ontology (GO) analysis was performed to characterize *CD24*-associated functions across three major categories: biological process, cellular component and molecular function. Biological process analysis indicated associations with cellular extravasation, leukocyte migration and cell–cell adhesion, as well as glomerular epithelial development and related epithelial differentiation processes. Cellular component analysis showed enrichment at the cell surface and plasma membrane, as well as other membrane-associated compartments. Molecular function analysis identified sialic acid binding, consistent with the involvement of CD24 in sialic acid-dependent molecular interactions. (Figure 1C). These findings, together with the STRING analysis identifying Siglec-10 within the CD24-associated protein interaction network (Figure 1B), provided the rationale for further investigating the CD24-Siglec-10 axis and its potential role in immune regulation.

### 3.2. mRNA and Protein Expression Analysis of CD24 in Multiple Cancers

**Figure 2:**
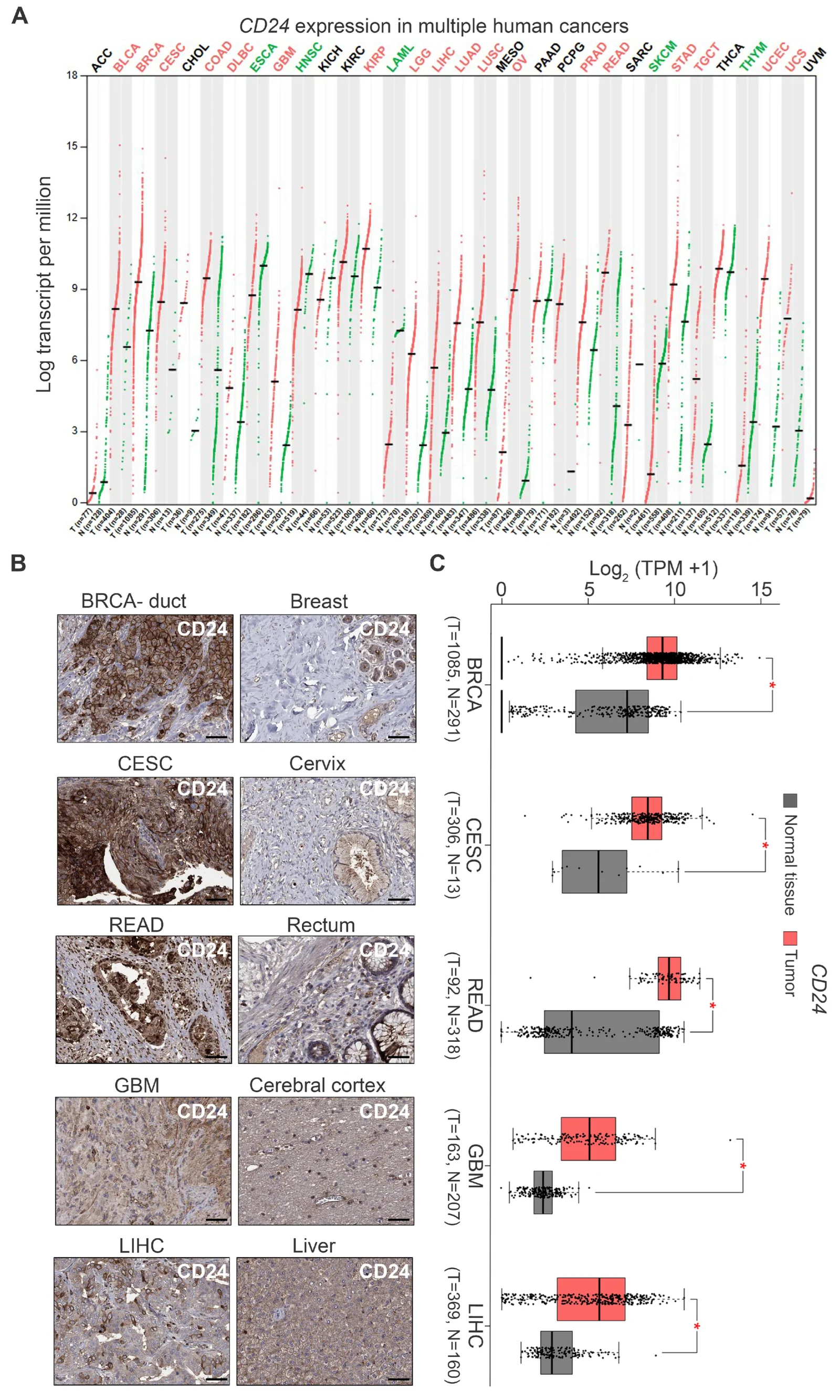
mRNA and protein expression of *CD24* in multiple human cancers. (**A**) Comprehensive mRNA expression analysis of *CD24* in various human cancers compared to normal tissue from TCGA database. (**B**) Immunohistochemistry images of significantly elevated expression of CD24 protein in BRCA, CESC, READ, GBM and LIHC compared to normal tissue. (**C**) Significantly elevated *CD24* mRNA expression in BRCA, CESC, READ, GBM and LIHC patients compared to normal tissue. Each dot represents the expression of each patient sample, where * indicates *p* ≤ 0.05 via one-way ANOVA test. T indicates tumor and N indicates normal tissue. The scale bar represents 50 µm.

To examine the transcriptional (mRNA) expression of *CD24* in multiple cancers, we used the TCGA database using the Gepia2.0 web tool. *CD24* mRNA expression was elevated in several cancers such as bladder cancer (BLCA), breast cancer (BRCA), cervical cancer (CESC), colorectal cancer (COAD), glioblastoma multiforme (GBM), liver cancer (LIHC), lung cancer (LUAD, LUSC), ovarian cancer (OV), rectal cancer (READ), and stomach cancer (STAD) (Figure 2A). To determine whether elevated CD24 expression was also evident at the protein level, we examined immunohistochemistry data from the Human Protein Atlas. Representative tumors and normal tissue sections demonstrated stronger CD24 immunostaining in breast cancer (BRCA), cervical cancer (CESC), rectal cancer (READ), glioblastoma multiforme (GBM), and liver cancer (LIHC) (Figure 2B, Table 1). We then compared *CD24* mRNA expression in these five tumors and determined significantly higher mRNA levels compared to normal tissue (Figure 2C). FPKM median expression of these cancers shows significant differences with a *p*-value of 2.0 × 10^−7^ (BRCA), 0.0018 (CESC), 0.00083 (READ), 0.028 (GBM), and 0.000082 (LIHC) (Table 1). Together, these analyses demonstrate that elevated *CD24* expression is observed across multiple human cancers and that, in selected tumor types, increased transcriptional expression is accompanied by stronger CD24 protein staining. These findings prompted us to further investigate the potential role of CD24 in tumor progression, metastasis, and regulation of the tumor immune microenvironment. 

### 3.3. Membrane-Associated CD24 Expression Correlates with Integrin β1 and SRC Expression in BRCA and CESC

**Figure 3:**
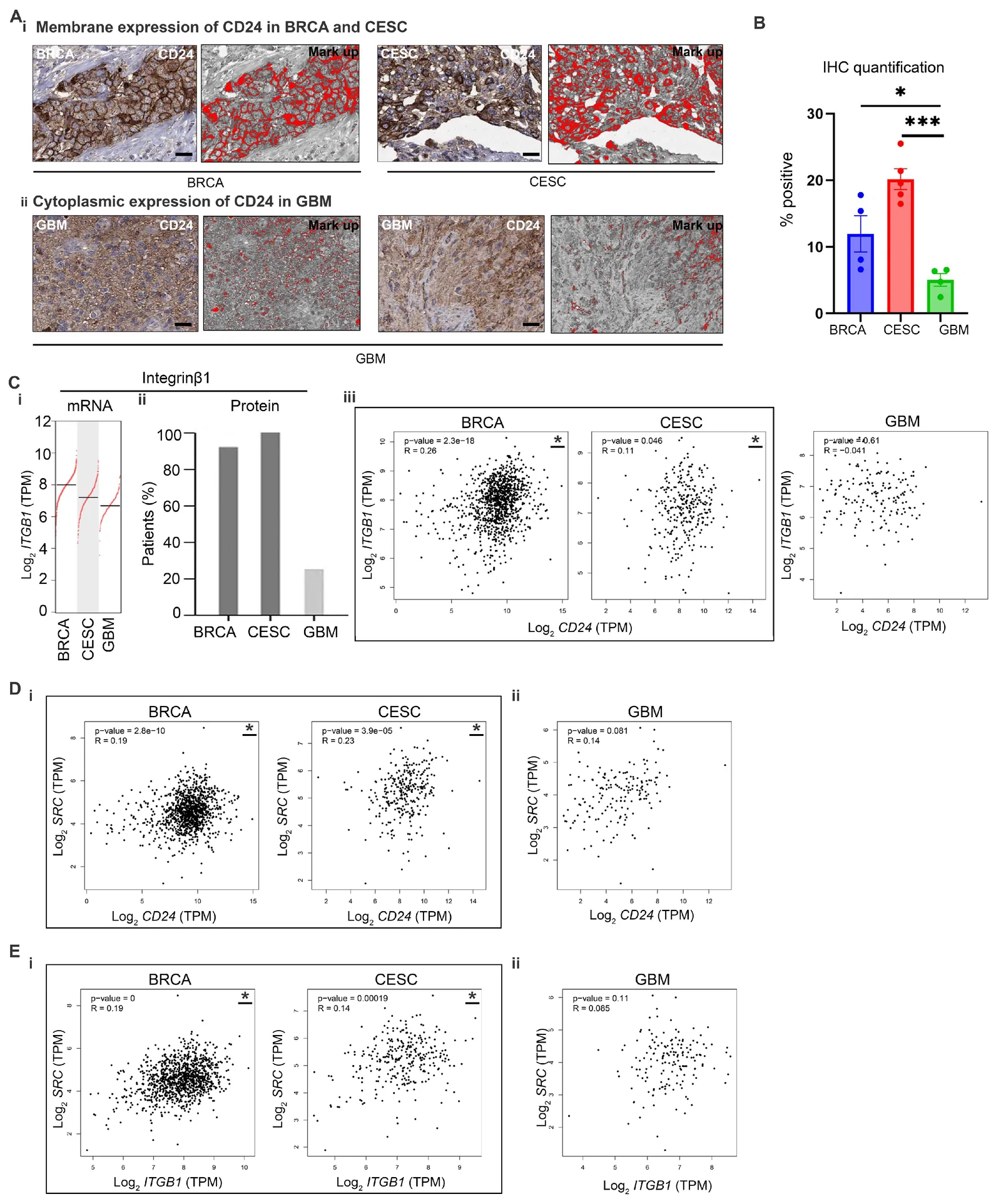
Association of membrane-localized CD24 with integrin β1/SRC expression in BRCA and CESC. (**A**) Representative immunohistochemistry (IHC) images of CD24 protein expression. For each tumor type, the left panel shows the original IHC image, while the right panel shows the corresponding markup image generated for image analysis, in which CD24-positive staining is highlighted in red and used for quantitative assessment. Panel (**i**) demonstrates predominantly membrane-associated CD24 staining in BRCA and CESC, whereas panel (**ii**) shows predominantly cytoplasmic CD24 staining in GBM. (**B**) Quantification of IHC images of CD24 expression in BRCA, CESC and GBM. (**C**) (**i**) mRNA expression of *ITGB1* and (**ii**) protein expression of integrin β1 as a percentage of patients expressing in BRCA, CESC and GBM. (**iii**) Pearson correlation analysis between mRNA expression of *CD24* and *ITGB1* in BRCA, CESC and GBM. (**D**) Spearman correlation analysis between mRNA expression of *CD24* and *SRC* in BRCA, CESC and GBM. (**E**) Kendall correlation analysis between mRNA expression of *ITGB1* and *SRC* in BRCA, CESC and GBM. Each dot represents the expression of each patient sample. * Indicates *p* ≤ 0.05 and *** indicates *p* ≤ 0.001 via (**C**–**E**) one-way ANOVA test and (**B**) *t*-test. The Scale bar represents 20 µm.

High expression of CD24 has been observed at the plasma membrane or in the cytosol. Although several studies have examined the cellular localization of CD24, the mechanisms that determine its localization within the cell are not yet fully understood. We therefore examined the cellular distribution of CD24 in BRCA, CESC and GBM. Analysis of CD24 immunohistochemistry staining obtained from the Human Protein Atlas (HPA) as well as recent research showed prominent membrane-associated staining in BRCA and CESC, whereas GBM showed predominant cytoplasmic staining [11,12,13,14] (Figure 3A). Quantification of CD24-positive staining further demonstrated differences among these tumor types (Figure 3B). Similarly, previous studies by Weichert et al. reported cytoplasmic localization of CD24 in colorectal cancer [15,16], while Deng et al. demonstrated both cytoplasmic and membrane CD24 staining in gliomas [17]. Because it was reported previously by Ruiz et al. that membrane-associated CD24 localizes within lipid rafts and recruits integrin β1 into these membrane domains [18], we decided to explore the relationship between CD24 and integrin β1 (ITGB1) further. *ITGB1* mRNA and integrin β1 protein expression were higher in BRCA and CESC than in GBM (Figure 3C(i,ii)). Correlation analysis further demonstrated a significant positive association between *CD24* and *ITGB1* expression in BRCA (R = 0.26, *p* = 2.3 × 10^−18^) and a weaker positive association in CESC (R = 0.11, *p* = 0.046), whereas no significant association was observed in GBM (R = −0.041, *p* = 0.61) (Figure 3C(iii)). These findings demonstrate an association between *CD24* and *ITGB1* expression in tumors displaying prominent membrane-associated CD24, consistent with the previously described relationship between membrane CD24 and integrin β1. Since it is well established that integrin β1 activates the FAK/SRC pathway [19,20,21], we decided to further examine correlations between *SRC* and both *ITGB1* and *CD24*. *SRC* and *CD24* expressions showed a significant positive correlation in BRCA (*p* = 2.8 × 10^−10^) and CESC (*p* = 3.9 × 10^−5^) (Figure 3D(i)). A weaker but statistically significant positive correlation was also observed in GBM (R = 0.14, *p* = 0.0181). Similarly, there was a significant positive correlation between *SRC* and *ITGB1* in BRCA and CESC (Figure 3E(i)), while this association was not statistically significant in GBM (Figure 3D(ii), E(ii)). Collectively, these findings identify coordinated expression of *CD24, ITGB1*, and *SRC*, particularly in BRCA and CESC, and are consistent with a potential relationship between membrane-associated CD24 and integrin β1/SRC signaling.

### 3.4. CD24 and Integrins-Associated TGFβ Signaling Network Activation in BRCA and CESC

**Figure 4:**
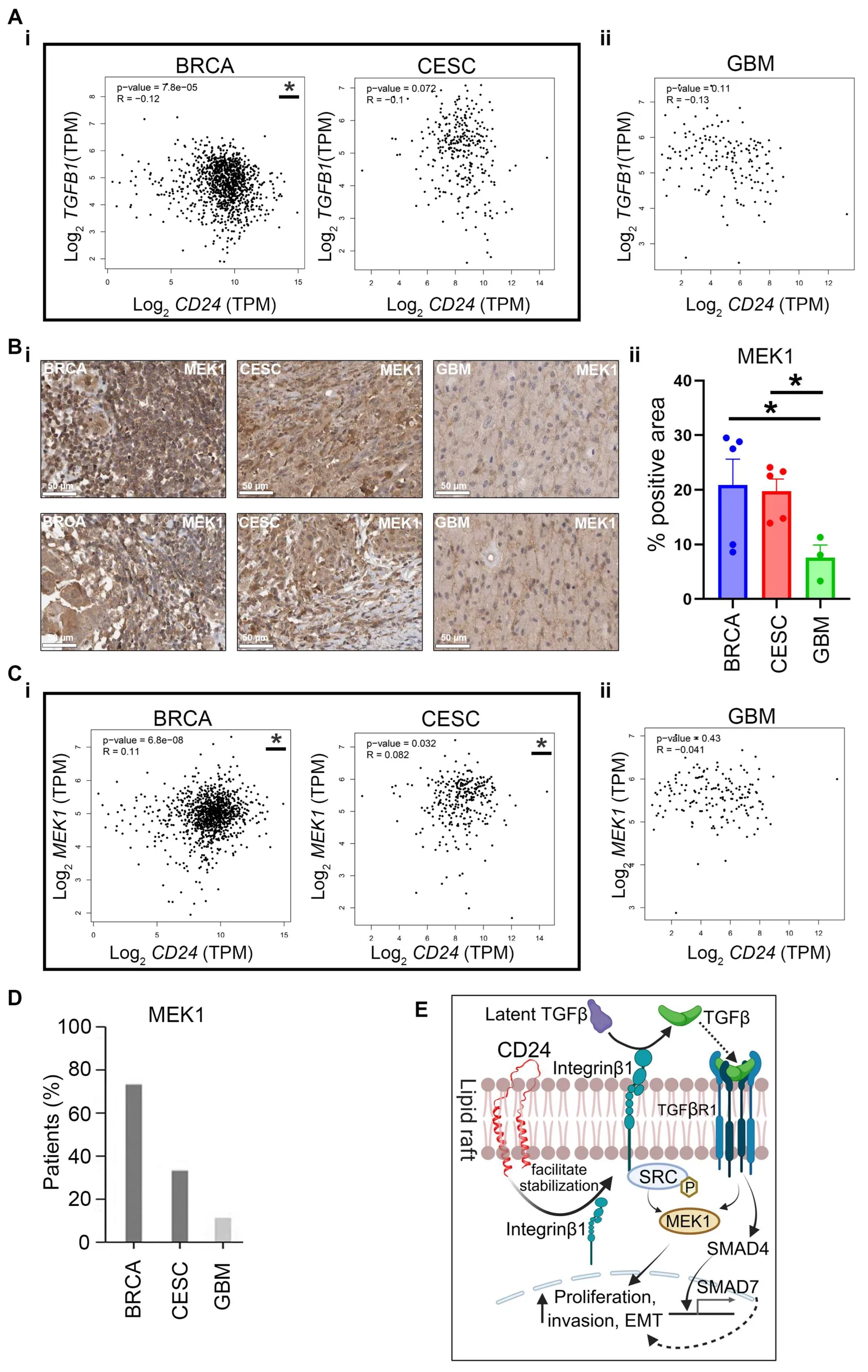
Membrane-localized CD24 and integrin β1 drive TGFβ1-mediated MEK1 activation. (**A**) Pearson correlation analysis between mRNA expression of *CD24* and *TGFB1* in (**i**) BRCA, CESC and (**ii**) GBM. (**B**) (**i**) Immunohistochemistry images of MEK1 protein expression and (**ii**) quantification of MEK1 protein expression in BRCA, CESC and GBM. (**C**) Correlation analysis between mRNA expression of *MEK1* and *CD24* in (**i**) BRCA, CESC and (**ii**) GBM. (**D**) Percentage of patients expressing MEK1. (**E**) Proposed mechanistic model integrating the present findings with previously published studies. The model summarizes the observed association of membrane-associated CD24 with integrin β1, TGFβ signaling, and MEK1 together with published evidence demonstrating CD24-mediated recruitment of integrin β1 into lipid rafts and integrin β1-dependent activation of TGFβ signaling. The downstream effects on proliferation, invasion, and epithelial-to-mesenchymal transition (EMT) represent biological processes previously reported in the literature and were not directly investigated in the present study. Each dot represents the expression of each patient sample. * Indicates *p* ≤ 0.05 via (**A**,**C**) one-way ANOVA test and (**B**(**ii**)) *t*-test. The scale bar represents 50 µm.

CD24-mediated stabilization of integrin β1 at lipid rafts plays an important role in the activation of multiple pathways involved in cell proliferation, invasion, migration, and metastasis [20]. One of the important roles of integrin β1 is to activate the TGFβ pathway by facilitating the conversion of latent TGFβ1 to its mature form [22,23,24], leading to activation of the canonical TGFβ/SMAD pathway [25] and noncanonical MEK1-associated signaling [26]. Additionally, conversion of latent TGFβ to its active form requires integrin β6 and integrin αV [27]. Our analysis showed higher *ITGB6* mRNA and integrin β6 protein expression, together with a positive correlation between *ITGB6* and *CD24* mRNA expression, in BRCA and CESC (Supplementary Figure S1A). We also observed abundant integrin αV protein expression and a significant positive Spearman correlation between *ITGAV* and *CD24* mRNA expression in BRCA and CESC (Supplementary Figure S1B). Moreover, TGFβ-mediated downstream activation plays a central role in immune suppression within the tumor microenvironment [28,29]. These observations prompted us to examine components of TGFβ signaling. To investigate associations with this pathway in BRCA, CESC, and GBM, we first performed Pearson correlation analysis between *CD24* and *TGFB1* mRNA expression. Pearson correlation analysis between *CD24* and *TGFB1* mRNA expression revealed a weak but statistically significant negative correlation in BRCA (R = −0.12, *p* = 7.8 × 10^−5^), whereas the correlations observed in CESC (R = −0.10, *p* = 0.072) and GBM (R = −0.13, *p* = 0.11) were not statistically significant (Figure 4A(i)). We further examined downstream components of TGFβ signaling, including SMAD4 and SMAD7. *SMAD7* mRNA and SMAD7 protein expression were higher in BRCA and CESC compared with GBM (Supplementary Figure S2A,B). We next examined MEK1, which is associated with noncanonical TGFβ signaling. Immunohistochemical analysis demonstrated greater MEK1 staining in BRCA and CESC than in GBM (Figure 4B(i)), and quantification confirmed significantly higher MEK1-positive areas in BRCA and CESC compared with GBM (Figure 4B(ii)). Consistent with these observations, correlation analysis demonstrated statistically significant positive associations between *CD24* and *MEK1* expression in BRCA (R = 0.11, *p* = 6.8 × 10^−8^) and CESC (R = 0.082, *p* = 0.032), whereas no significant association was observed in GBM (R = −0.041, *p* = 0.43) (Figure 4C). Analysis of patient samples further showed that MEK1 protein expression was detected in a substantially greater proportion of BRCA (~75%) and CESC (~37%) samples than GBM (~10%) samples (Figure 4D). MEK1 overexpression plays a prominent role in tumorigenesis associated with proliferation, tumor invasion, EMT and metastasis [30,31]. Collectively, these findings demonstrate an association between *CD24* expression and components of the integrin β1/TGFβ signaling network, particularly in BRCA and CESC. Together with previous evidence that membrane-associated CD24 can recruit integrin β1 to lipid rafts and that integrin β1 can contribute to TGFβ activation [14,16,17,18], our findings support a proposed model in which membrane-associated CD24 may facilitate integrin β1-dependent signaling, potentially contributing to downstream TGF-β/SMAD and MEK1-associated signaling pathways (Figure 4E). 

### 3.5. Membrane-Localized CD24-Siglec-10 Interaction Is Associated with Immune Suppression in BRCA and CESC

**Figure 5:**
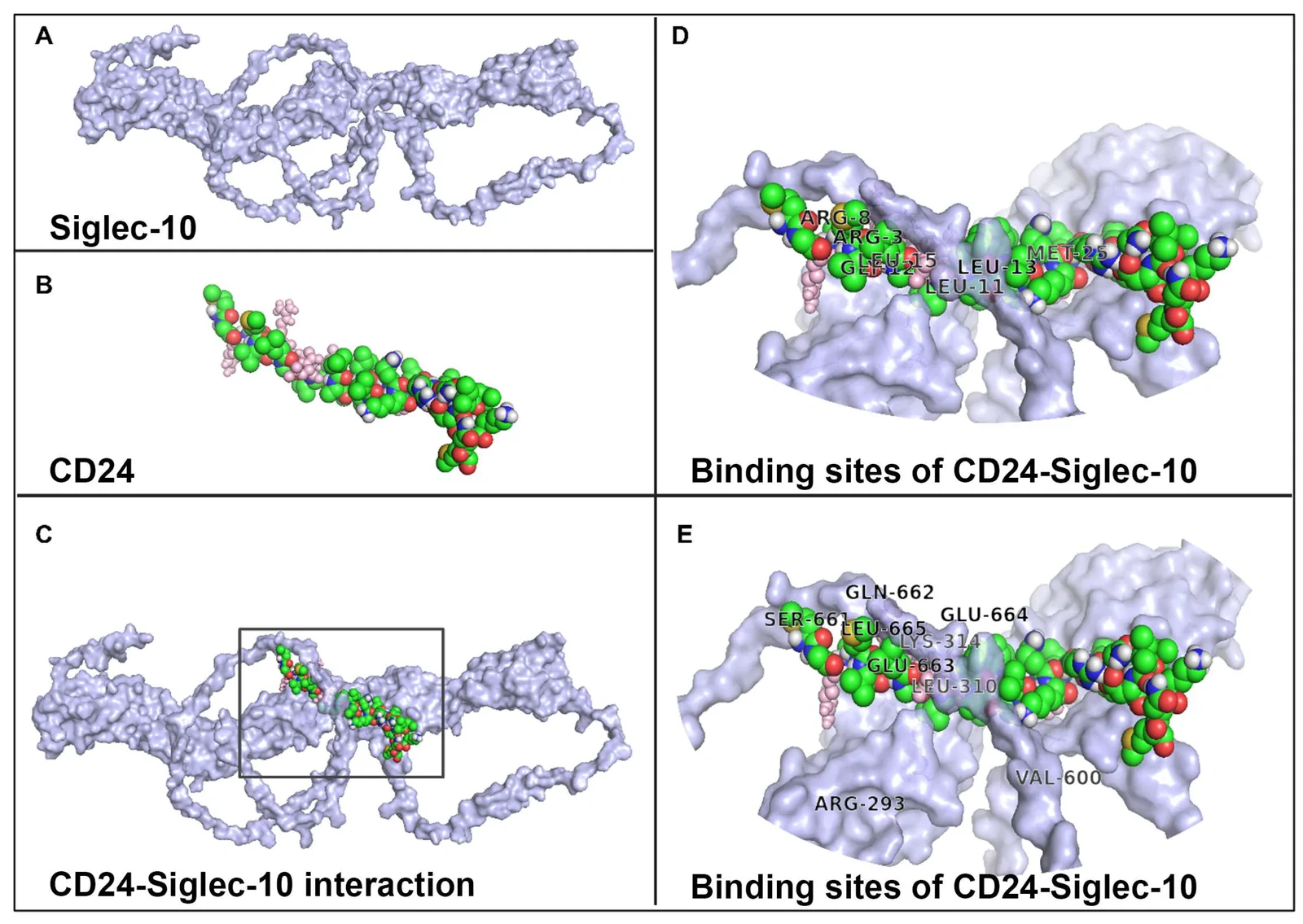
Structural docking model of CD24 interaction with Siglec-10. (**A**) Surface representation of the Siglec-10 ectodomain used for docking analysis. (**B**) Space-filling representation of the CD24 peptide used in the docking simulations. (**C**) Overall docking model of CD24 bound to Siglec-10. CD24 is shown as a space-filling model positioned within a shallow binding groove on the surface of Siglec-10. The boxed region highlights the predicted binding interface between the two molecules. (**D**) Close-up view of the CD24 interaction hotspot identified in docking simulations. Key residues within the CD24 peptide predicted to participate in binding include Arg3, Arg8, Leu11, Gly12, Leu13, Leu15, and Met25, forming the core interaction region within the Siglec-10 binding pocket. (**E**) Close-up of Siglec-10 residues predicted to contribute to the interaction with CD24. Residues surrounding the binding interface include Ser661, Gln662, Glu663, Glu664, Leu665, Lys314, Leu310, Val600, and Arg293, which collectively form the electrostatic and hydrophobic environment accommodating the CD24 peptide. Docking models were generated using ClusPro protein–protein docking software and visualized in PyMOL to identify residues located within the predicted binding interface.

The CD24 protein interaction network shown in Figure 1B identified Siglec-10, an inhibitory receptor expressed by immune cells, as a potential CD24-interacting protein. Previous studies by G. Chen et al. and J. Kopecky et al. experimentally demonstrated the interaction between CD24 expressed on cancer cells and Siglec-10 expressed on tumor-associated macrophages (TAM) using immunoprecipitation and immunofluorescence approaches [14,32]. These findings prompted us to further explore the potential structural basis of this established interaction using protein–protein docking, as the three-dimensional binding interface between CD24 and Siglec-10 has not yet been experimentally resolved. Protein–protein docking was performed using the ClusPro web server between CD24 as a receptor and Siglec-10 as a ligand (Figure 5A–C). Analysis of the predicted docking complexes identified a conserved CD24-Siglec-10 binding interface involving both electrostatic and hydrophobic interactions. Charged amino acids at the N-terminus of the CD24 protein help attract Siglec-10, while the middle part of CD24, the main binding site, forms tight hydrophobic contacts that stabilize the binding. The main CD24 binding site consistently interacts with the same region of the Siglec-10 ectodomain, which forms two separate interaction patches. The first patch is enriched in acidic and polar amino acid residues of Siglec-10, most notably Glu663, Glu664, Glu660, Ser661, and Gln662, which interact with Arg3 and Arg8 on CD24, supporting a stable electrostatic and hydrogen-bonding interface (Figure 5D,E). The second patch contains predominantly hydrophobic or mixed-character residues such as Arg293, Leu310, Pro311, Lys314, Leu665, Val600, and His666. These residues of Siglec-10 repeatedly interact with the Leu11-Leu15 segment and Met25 of CD24. These interactions form a hydrophobic packing surface that helps stabilize the orientation of the CD24 peptide within the binding pocket of Siglec-10 (Figure 5D,E). Taken together, these results show that interaction of CD24 with Siglec-10 is driven by cooperative electrostatic anchoring of the N-terminal residues of CD24 to acidic regions of Siglec-10, combined with hydrophobic packing of the central leucine-rich segment, which produces a structurally consistent and energetically stable interaction. 

**Figure 6:**
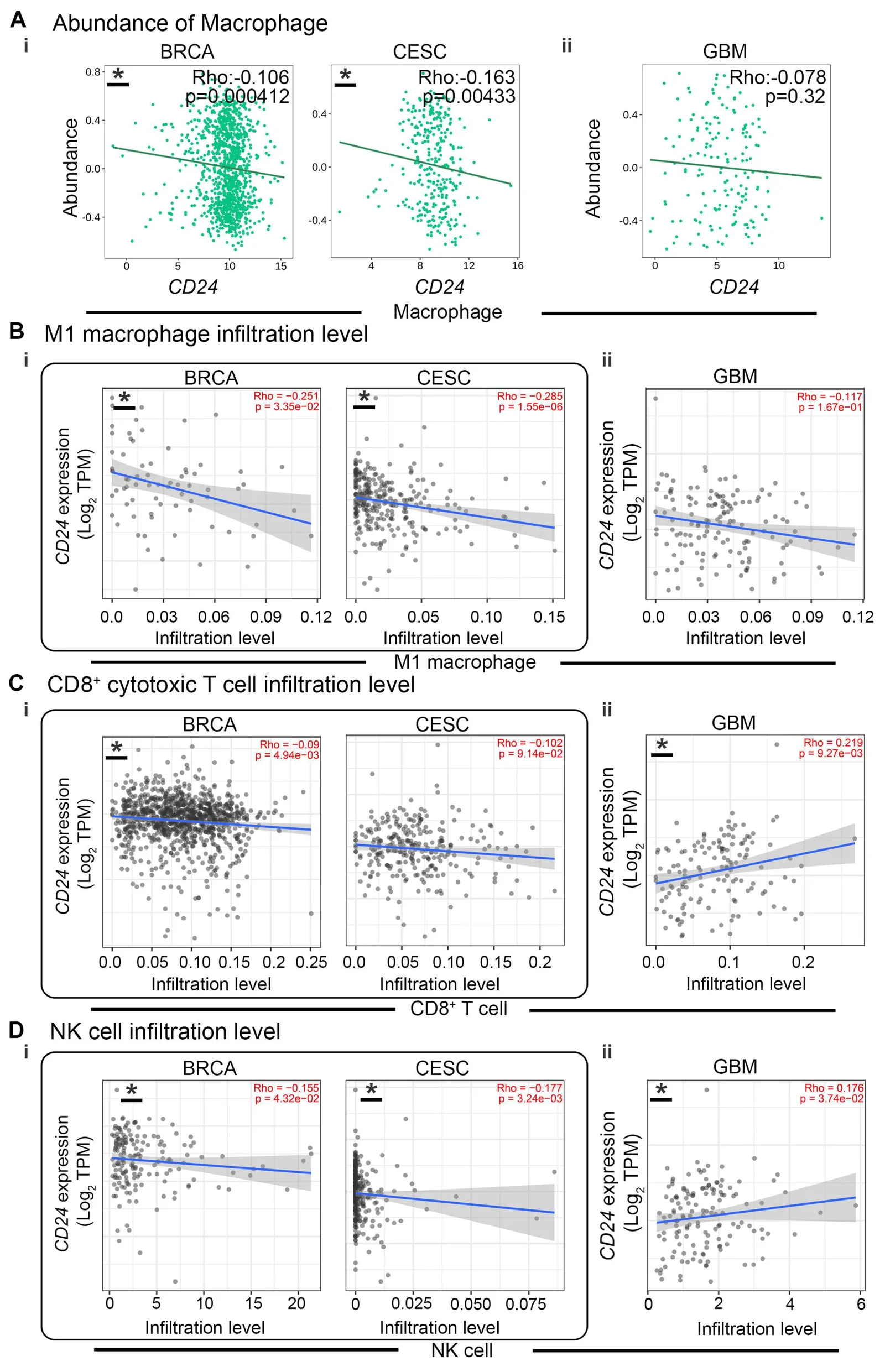
Association between *CD24* expression and macrophage abundance and infiltration of M1 macrophages, CD8^+^ T-cells and NK cells in BRCA, CESC, and GBM. (**A**) Abundance of macrophages with *CD24* expression in (**i**) BRCA, CESC and (**ii**) GBM. (**B**–**D**) Scatter plot of relationship between *CD24* expression and infiltration of (**B**) M1 macrophages, (**C**) CD8^+^ T-cells, and (**D**) NK cells in (**i**) BRCA, CESC and (**ii**) GBM. Each dot represents the expression of each patient sample. * Indicates *p* ≤ 0.05 via (**A**–**D**) one-way ANOVA test.

Engagement of Siglec-10 on macrophages by CD24 expressed on tumor cells has been reported to recruit the inhibitory phosphatases SHP-1 and SHP-2 and suppress macrophage-mediated antitumor activity and phagocytosis [33]. We therefore examined if *CD24* expression was associated with macrophage abundance and M1 macrophage infiltration in BRCA, CESC, and GBM. *CD24* expression was significantly negatively associated with macrophage abundance in BRCA and CESC. In contrast, no significant association was observed in GBM (Figure 6A). Similarly, *CD24* expression was negatively associated with estimated M1 macrophage infiltration in BRCA and CESC, whereas the association in GBM was not statistically significant (Figure 6B). Previous studies have implicated the CD24-Siglec-10 axis in the regulation of adaptive and innate antitumor immunity. Blockade of CD24-Siglec-10 signaling has been reported to enhance CD4^+^ and CD8^+^ T-cell responses and activate T-cell receptor (TCR)- and B-cell receptor (BCR)-related kinases, thus inhibiting tumor immune escape [34]. Additionally, a blockade of this interaction increased NK-cell cytotoxicity, promoting direct killing of cancer cells [35]. Following these observations, we examined the correlation between *CD24* expression and infiltration of CD8^+^ T-cells and NK cells. Our purity-adjusted data revealed that estimated infiltration of CD8^+^ cytotoxic T-cells and NK cells was negatively associated with *CD24* expression in BRCA and CESC; a significant positive association was observed in GBM (Figure 6C,D, Supplementary Figures S3–S5). Together, these findings demonstrate clear relationships between *CD24* expression and immune-cell infiltration across these tumor types. In BRCA and CESC, the association between elevated *CD24* expression and reduced infiltration of M1 macrophages, CD8^+^ T-cells, and NK cells is consistent with an immunosuppressive tumor microenvironment and supports a potential role for the CD24-Siglec-10 immune checkpoint axis in immune evasion.

### 3.6. Difference in Subcellular Localization of CD24 Affects the Prognosis in BRCA, CESC and GBM

**Figure 7:**
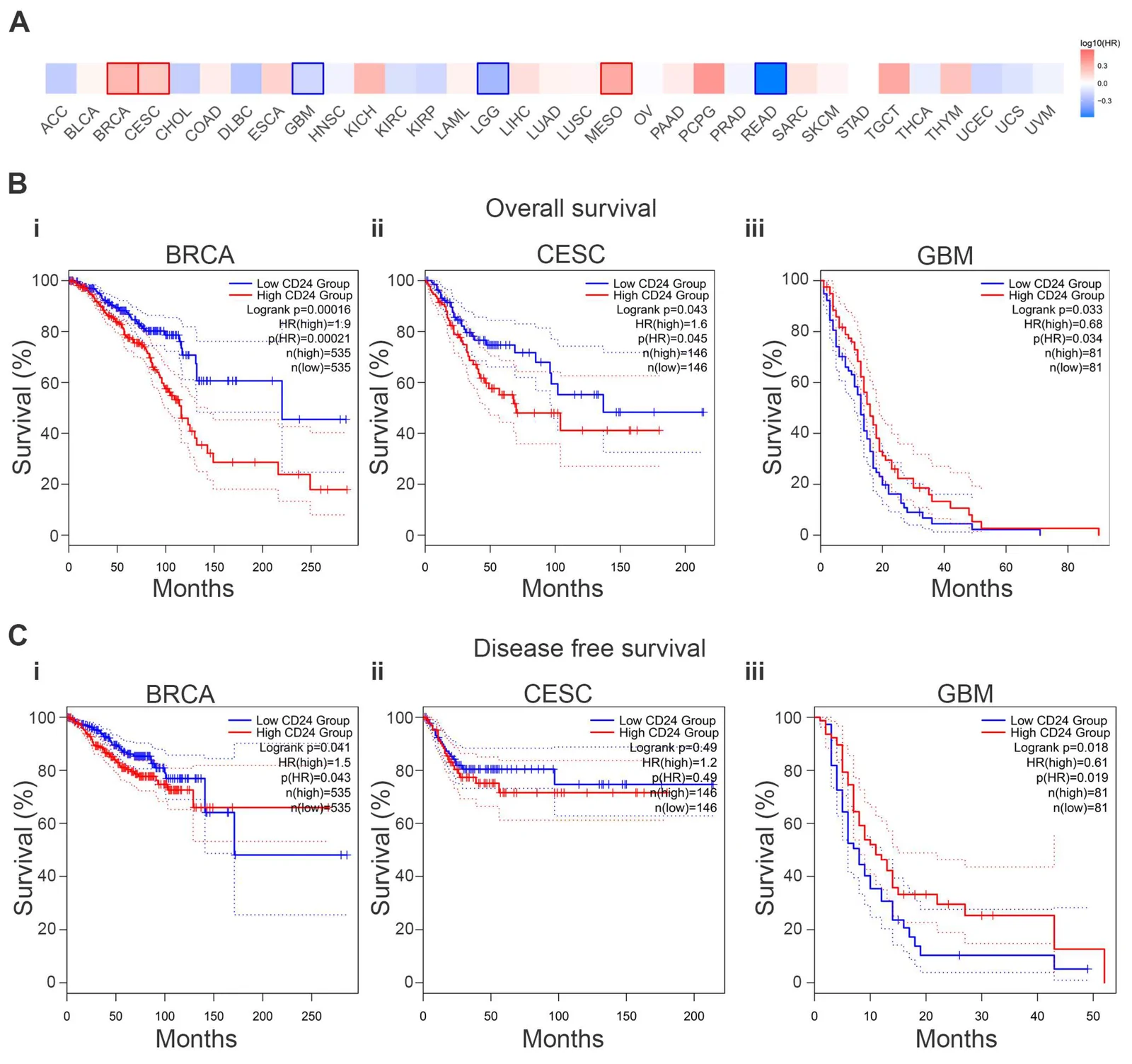
*CD24* expression-associated prognostic analysis in BRCA, CESC and GBM. (**A**) Survival contribution map of *CD24* using the Mantel–Cox test, red indicates higher risk and blue indicates lower risk. (**B**) Overall survival based on high vs. low expression of *CD24* using the Kaplan–Meier analysis in (**i**) BRCA, (**ii**) CESC and (**iii**) GBM. (**C**) Disease-free survival based on high vs. low expression of *CD24* using the Kaplan–Meier analysis in (**i**) BRCA, (**ii**) CESC and (**iii**) GBM.

Our previous analyses demonstrated distinct patterns of CD24 localization and associated signaling among BRCA, CESC, and GBM. BRCA and CESC exhibited prominent membrane-associated CD24 expression together with increased integrin β1 expression and associations with components of TGFβ-related signaling and an immunosuppressive tumor microenvironment. In contrast, CD24 staining in GBM was predominantly cytoplasmic and was associated with a distinct signaling and immune-infiltration profile. Based on these differences, we next investigated whether *CD24* expression was associated with patient prognosis in these tumor types. Survival contribution mapping of *CD24* using Mantel–Cox test revealed that elevated *CD24* expression in BRCA and CESC was associated with high risk, whereas the opposite trend was observed in GBM (Figure 7A). Kaplan–Meier analysis further demonstrated that high *CD24* expression was significantly associated with poorer overall survival in BRCA (HR = 1.9, *p* = 0.00021) and CESC (HR = 1.6, *p* = 0.045) (Figure 7B(i,ii)). In contrast, high *CD24* expression in GBM was associated with a favorable outcome (*p* = 0.034) (Figure 7B(iii)). Disease-free survival showed a similar overall trend, with high *CD24* expression associated with poorer disease-free survival in BRCA and improved disease-free survival in GBM, while the association in CESC was not statistically significant (Figure 7C). Collectively, these findings demonstrate that the prognostic significance of *CD24* is tumor-type dependent. High *CD24* expression is associated with unfavorable overall survival in BRCA and CESC, whereas the opposite relationship is observed in GBM. When considered together with our previous observations regarding subcellular CD24 protein localization, these findings suggest that differences in CD24 localization may contribute to distinct biological functions and prognostic associations across these tumor types. 

## 4. Discussion

CD24 was initially identified as a B-cell differentiation antigen, expressed across a broad spectrum of tissues, including both hematopoietic and non-hematopoietic tissues [36]. The broad immune expression pattern of CD24 includes its expression on B-cells, T-cells and macrophages. In B-cells, it participates in the homeostatic regulation of B-cell development and activation [37], whereas on T lymphocytes, it plays a critical role in providing co-stimulatory signals necessary for clonal expansion and proliferation [38]. The interaction between CD24 and Siglec-10 on macrophages has been shown to confer an antiphagocytic effect. Additionally, CD24 interacts with L-selectin and P-selectin to facilitate rolling and trafficking of leukocytes into inflamed tissues. Concurrently, CD24 functions as a suppressor of various damage-associated molecular patterns (DAMPs), such as high mobility group box-1 (HMGB1), thereby preventing their overactivation and consequent heightened inflammatory responses. These protective mechanisms also contribute to safeguarding against autoimmunity. Our results confirm this fact, as STRING analysis revealed interactions between CD24 and molecules such as SELP (P-selectin), SIRPA, HMGB1, Siglec-10, and CD44, which are critical immune checkpoints, cellular markers, and danger signals involved in orchestrating immune responses in inflammation and cancer.

CD24 is frequently overexpressed in various solid tumors and hematologic malignancies, where it contributes to tumorigenesis by regulating cancer stem cell activity, tumor proliferation, metastasis, and immune evasion [39]. Moreover, recent research indicates CD24 as an independent biomarker in ovarian cancer [40], breast cancer [41,42], colorectal cancer [43], cervical cancer [44], and cholangiocarcinoma [45]. Similarly, our results show high mRNA and protein expression of *CD24* in BRCA, CESC, READ, GBM and LIHC compared to the normal counterpart. Exploring CD24’s role in maintaining cancer stem-like cell properties, Y. Jang et al. demonstrated that CD24 regulates the expression of miR-130a and miR-301a via STAT4 and YY1 phosphorylation-mediated SRC and FAK activation; they induce chemoresistance and a cellular quiescence-like state in ovarian cancer [46]. Additionally, T. Lee et al. found that CD24^+^ HCC cells drive tumor-initiating cell (T-IC) genesis through STAT3-mediated NANOG regulation, which was found to be critical for the maintenance, self-renewal, differentiation, and metastasis of tumors, and to impact patients’ clinical outcomes [47]. These results suggest CD24’s involvement in intracellular tumor proliferation, metastasis and stemness maintenance pathways. Our analysis revealed prominent membrane-associated CD24 localization in BRCA and CESC, consistent with its reported localization within membrane lipid rafts, whereas CD24 in GBM was predominantly localized to the cytoplasm. Baumann and associates reported that lipid raft-localized, membrane-bound CD24 recruits integrin β1 (ITGB1) into raft domains and promotes c-Src activity within lipid rafts to enhance integrin-dependent adhesion [19]. Consistent with these previous mechanistic observations, BRCA and CESC displayed higher *ITGB1* expression than GBM, and *CD24* expression positively correlated with *ITGB1* expression in both tumor types. Furthermore, *CD24* and *ITGB1* each showed positive associations with *SRC* expression in BRCA and CESC. Although these expression-based analyses do not establish pathway activation or direct recruitment of integrin β1 into lipid rafts, they are consistent with the previously described CD24-integrin β1-SRC relationship and support its further investigation in these tumor types. 

Integrin β1 is a well-known mediator of latent TGFβ activation through interaction with the latency-associated peptide [22] and activation of TGFβ promotes tumor progression through EMT, immune suppression/evasion, angiogenesis and ECM remodeling by activation of the canonical SMAD-dependent pathway or non-canonical pathways such as MAPK, ERK, MEK1, and PI3K/AKT [48]. In our analysis, *CD24* and *TGFB1* transcript levels were not positively correlated. Instead, a weak but statistically significant negative correlation was observed in BRCA, while the associations in CESC and GBM were not statistically significant. Thus, transcript-level correlations do not support direct activation of TGFβ1 expression by CD24. Nevertheless, BRCA and CESC exhibited distinct expression patterns of several components associated with TGFβ signaling compared with GBM. *SMAD7* mRNA and SMAD7 protein expression was particularly elevated in BRCA and, to a lesser extent, CESC, compared with GBM, whereas *SMAD4* mRNA and SMAD4 protein expressions did not show the same consistent pattern. In addition, MEK1 protein expression was substantially higher in BRCA and CESC than in GBM, supporting differences in non-canonical TGFβ-associated signaling among these tumor types. Collectively, these findings are consistent with differential engagement of TGFβ-associated signaling networks rather than direct transcriptional regulation of *TGFβ1* by *CD24*. When considered together with the distinct CD24 localization patterns observed in these cancers and previously reported functions of lipid raft-associated CD24, our findings support the possibility that membrane-associated CD24 may be associated with differences in these signaling networks beyond *CD24* expression levels alone.

The biological interaction between CD24 and Siglec-10 is well established and contributes to tumor evasion of innate immune surveillance via suppression of macrophage-mediated phagocytosis. Based on this established biological interaction, we performed a protein–protein docking analysis to predict the structural interface between CD24 and Siglec-10, which shows a structurally consistent and energetically stable interaction. Furthermore, in line with the previously reported CD24-Siglec-10-SHP1/SHP2 pathway that mediates suppression of macrophage M1 polarization and phagocytic activity [8], our data revealed reductions in M1 macrophage infiltration associated with high *CD24* expression in breast cancer (BRCA) and cervical squamous cell carcinoma (CESC), but not in glioblastoma. A similar trend was observed for cytotoxic CD8^+^ T-cells and natural killer (NK) cell infiltration, with *CD24* expression negatively correlating with these immune cell populations in BRCA and CESC but not in GBM. 

**Figure 8:**
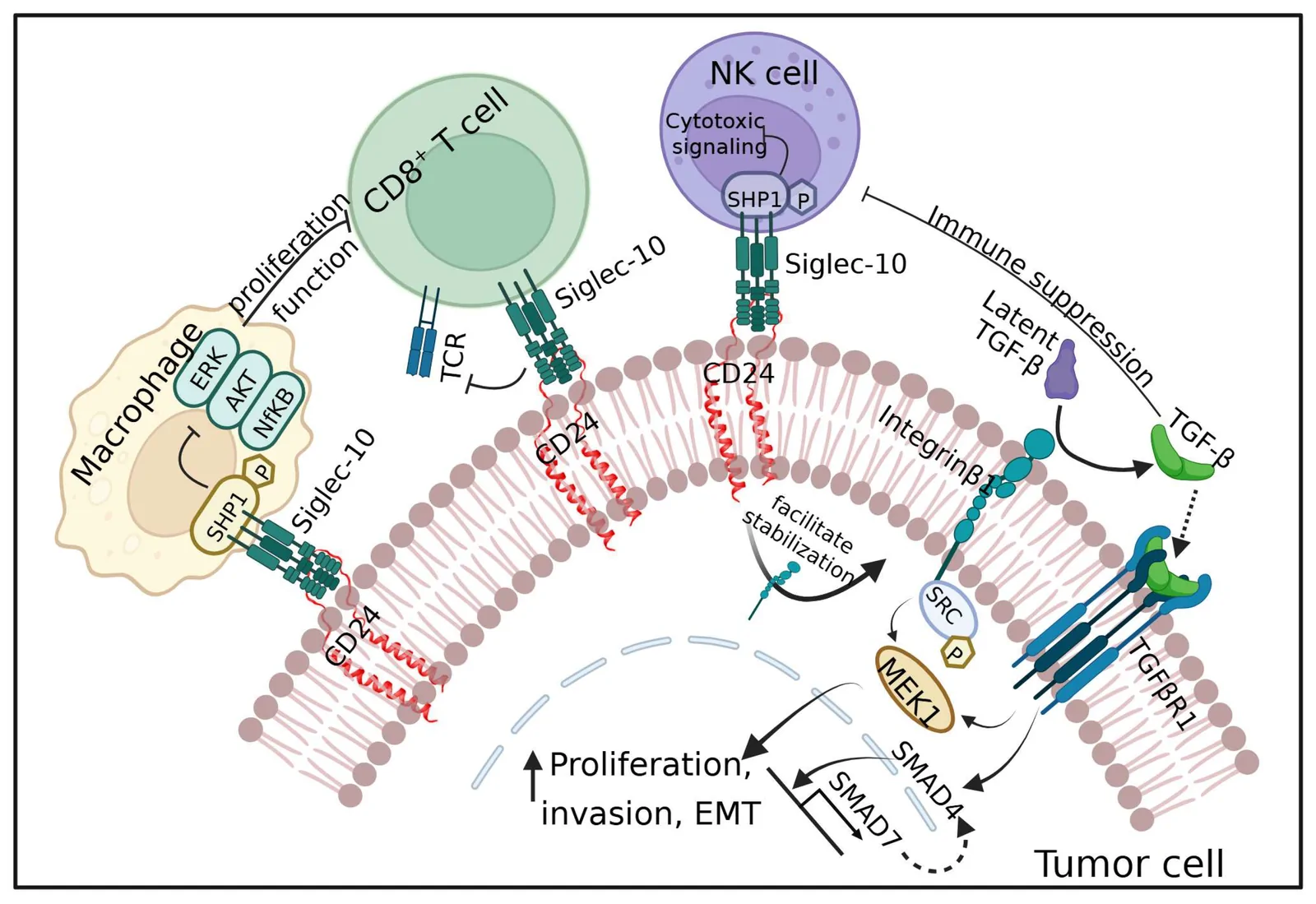
Proposed model of membrane-localized CD24 involvement in intracellular signaling and tumor immune regulation. Membrane-associated CD24 is proposed to be associated with integrin β1/TGFβ/MEK1 signaling and with Siglec-10-mediated macrophage immune suppression in the tumor microenvironment of BRCA and CESC. Reduced infiltration of M1 macrophages, CD8^+^ T-cells, and NK cells associated with elevated *CD24* expression is incorporated into the model based on the present immune-infiltration analyses.

Taken together, our findings suggest that subcellular localization may refine CD24 beyond an abundance-based biomarker, potentially distinguishing which CD24-high tumors are most likely to harbor an active ITGβ1/TGFβ-driven, immune-suppressed phenotype. This proposed interaction led us to draw a model that applies to BRCA and CESC and predicts the poor clinical outcome in them (Figure 8). 

## 5. Conclusions

Overall, our comprehensive analysis identifies membrane-associated CD24 as a potential link between tumor-promoting signaling and immune evasion in BRCA and CESC. Membrane-associated CD24, potentially through its localization within lipid rafts, was associated with the integrin β1/TGFβ/MEK1 signaling axis, while structural modeling and protein–protein docking revealed a consistent and energetically favorable interface between CD24 and the immune checkpoint receptor Siglec-10. These molecular features were accompanied by an immunosuppressive immune-infiltration profile and unfavorable clinical outcomes, suggesting that the subcellular localization of CD24 may be an important determinant of its tumor-promoting functions. 

## 6. Limitations and Future Directions

These promising data were mainly obtained from the patient database of the Cancer Genome Atlas. While it represents actual human data, the TCGA database exhibits demographic limitations as it underrepresents certain ethical and racial groups compared to the US population. Moreover, the TCGA database also has clinical limitations as it represents incomplete or missing information about treatment history, stage of disease, or follow-up details. An additional limitation of this study is that the immunohistochemical localization analyses (Human Protein Atlas) and transcriptomic correlation analyses (TCGA/GTEx) were performed using independent patient cohorts. Although the findings from these complementary datasets consistently support the proposed biological model, future studies integrating protein localization and transcriptomic analyses in matched patient samples will further strengthen these observations. 

## Figures and Tables

**Table 1:** Statistical analysis of the expression of CD24 in multiple cancers along with % of 5-year survival data and significance.

Cancer Type	FPKM Median Expression	*p* Value	% of 5-Year Survival with High Expression	% of 5-Year Survival with Low Expression	Protein Expression	Hazard Ratio (HR)	*p* (HR)	Significance	Condition
**Breast cancer**	**755.83**	**2.0 × 10^−7^**	**75**	**89**	**High**	**1.9**	**0.00021**	**Prognostic**	**Unfavorable**
**Cervical cancer**	**531.43**	**0.0018**	**54**	**79**	**high**	**1.6**	**0.045**	**Prognostic**	**Unfavorable**
**Rectal cancer (READ)**	**972.02**	**0.00083**	**67**	**0**	**High/medium**	**0.27**	**0.023**	**Prognostic**	**Favorable**
Endometrial cancer	1055.6	0.31	65	63	High/medium	0.69	0.29	Not prognostic	
**Glioblastoma (GBM)**	**39.88**	**0.028**	**15**	**3**	**Medium**	**0.68**	**0.034**	**Potential prognostic**	**Favorable**
Head and neck cancer	336.35	0.076	47	43	Medium	0.89	0.38	Not prognostic	
Liver cancer	57.95	0.000082	36	53	Medium/ low	1.3	0.12	Not prognostic	
Lung adenocarcinoma	232.26	0.19	42	37	High/medium	1.1	0.45	Not prognostic	
Lung squamous cell carcinoma	230.12	0.44	46	49	Medium	1.1	0.48	Not prognostic	
Melanoma cancer	3.69	0.24	37 (3 yr)	45 (3 yr)	Low	1.1	0.47	Not prognostic	
Ovarian cancer	613.04	0.31	29	37	High	0.96	0.77	Not prognostic	
Pancreatic cancer	449	0.023	22	44	High/medium	1.2	0.42	No prognostic	
Prostate cancer	241.02	0.09	97	99	High/medium	0.91	0.88	Not prognostic	
Renal clear cell carcinoma	1384.18	0.019	70	56	High/medium	0.74	0.056	Not prognostic	
Renal papillary cell carcinoma	2381.15	0.095	77	72	Medium	0.68	0.22	Not prognostic	
Stomach cancer	693.18	0.29	33	42	High/medium	1	0.95	Not prognostic	
Bladder Urothelial Carcinoma	412.25	0.16	6	0	High/medium	1.1	0.54	Not prognostic	

## Data Availability

The original contributions presented in this study are included in the article/Supplementary Material. Further inquiries can be directed to the corresponding author.

## Supplementary Materials

The following supporting information can be downloaded at: https://www.mdpi.com/article/10.3390/biology15181611/s1, Supplementary Figure S1: (A) Integrin β6 and (B) Integrin αV mRNA and protein expression together with its association with CD24. Supplementary Figure S2: TGFβ-mediated Smad activation in BRCA and CESC. Supplementary Figure S3: M1 Macrophage infiltration level and its association with CD24 expression, adjusted to tumor purity. Supplementary Figure S4: CD8+ cytotoxic T cell infiltration level and its association with CD24 expression, adjusted to tumor purity. Supplementary Figure S5: NK cell infiltration level and its association with CD24 expression, adjusted to tumor purity.
