# Supplementary material for: Subcellular Localization of CD24 Is Associated with Integrin β1 Signaling, Immune Suppression, and Clinical Outcomes Across Human Cancers
Source: Biology (Basel). 2026 Sep 12;15(18):1611. doi: 10.3390/biology15181611 (PMC13604725; doi:10.3390/biology15181611)
Supplement: Supplementary file 1 [file biology-15-01611-s001.zip › biology-4503345-supplementary.pdf]

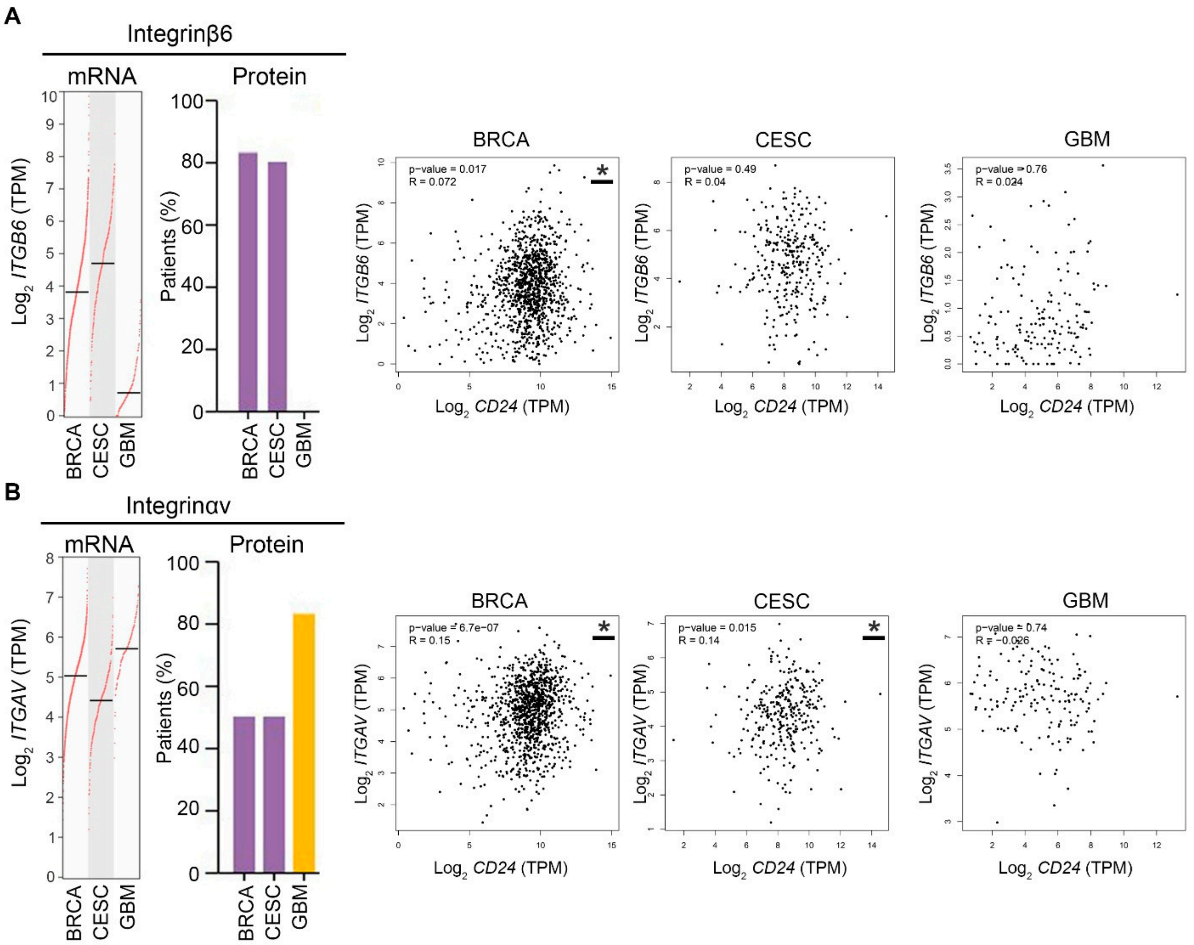

Supplementary Figure S1: (A) Integrin β6 and **(B)** Integrin αV mRNA and protein expression together with its association with CD24. Protein expression is presented as % of patients expressing each protein.

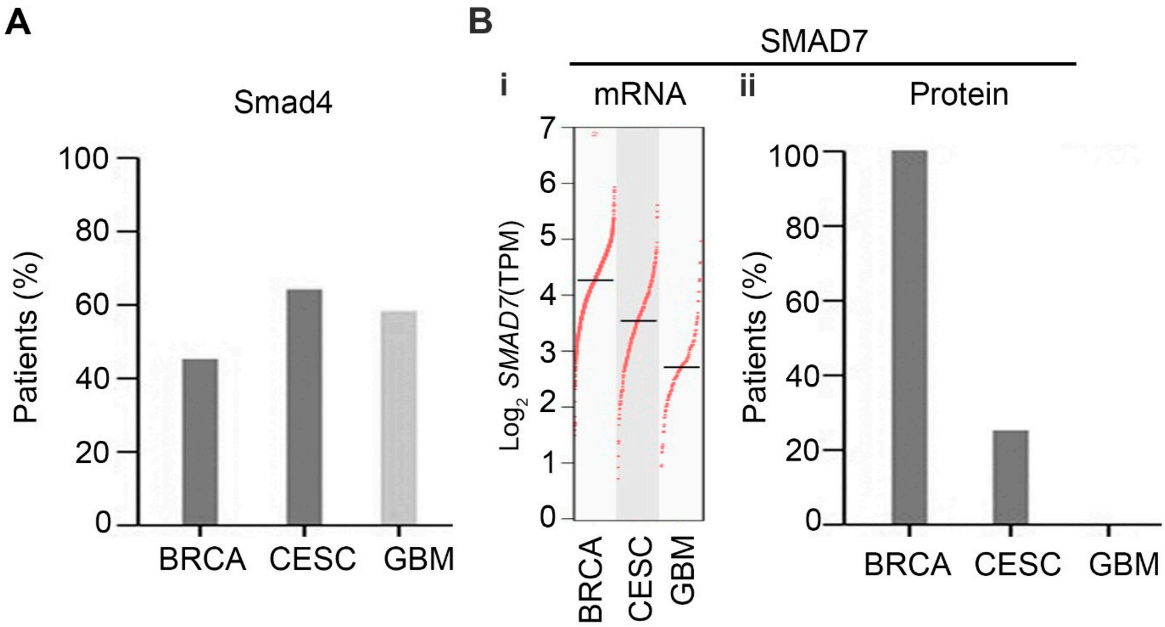

Supplementary Figure S2: TGFβ-mediated Smad activation in BRCA and CESC. **(A)** Percentage of patients expressing Smad4 protein. **(B) (i)** mRNA expression of SMAD7 and **(ii)** protein expression of Smad7 as a percentage of patients expressing in BRCA, CESC and GBM.

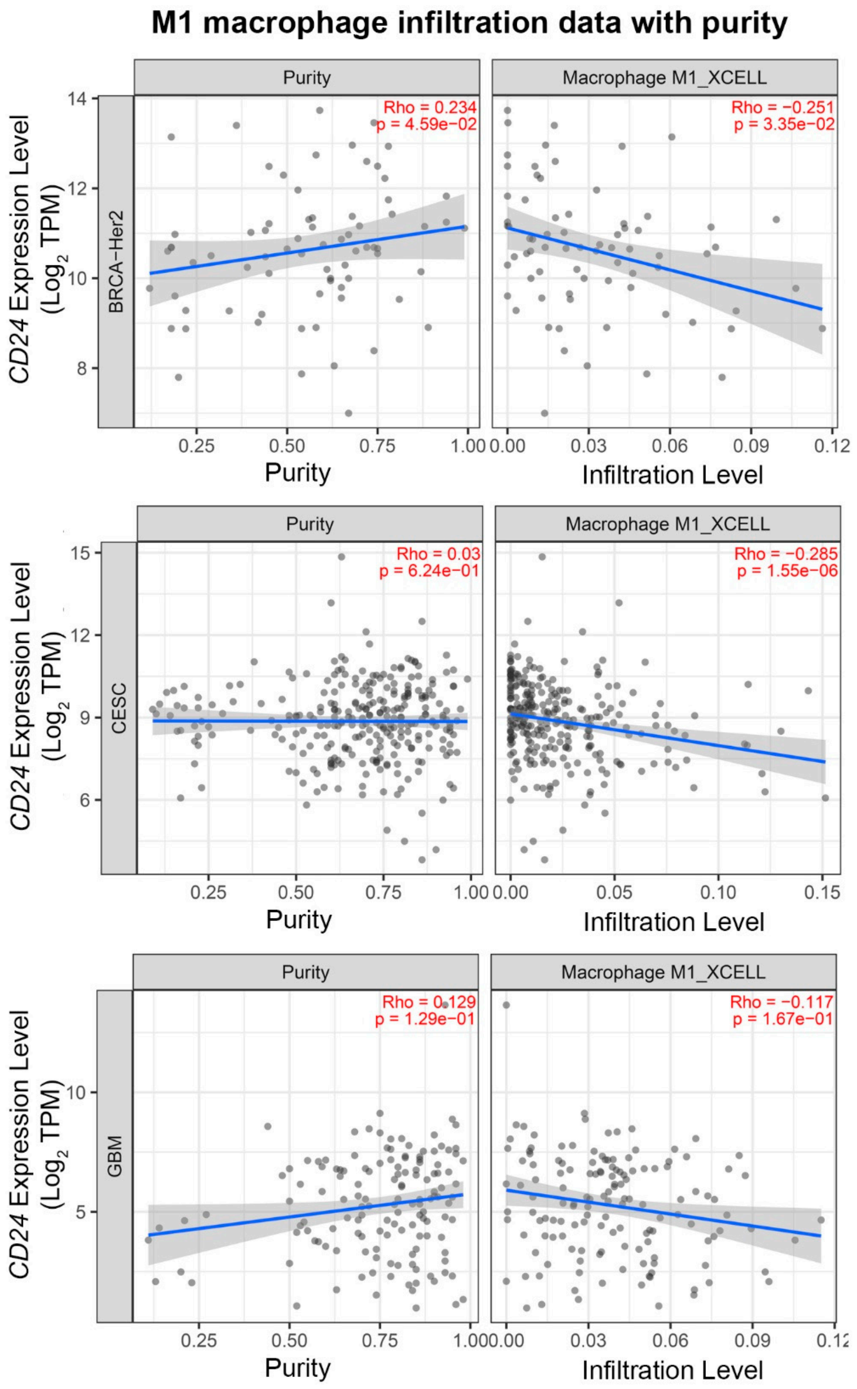

Supplementary Figure S3: M1 Macrophage infiltration level and its association with CD24 expression, adjusted to tumor purity.

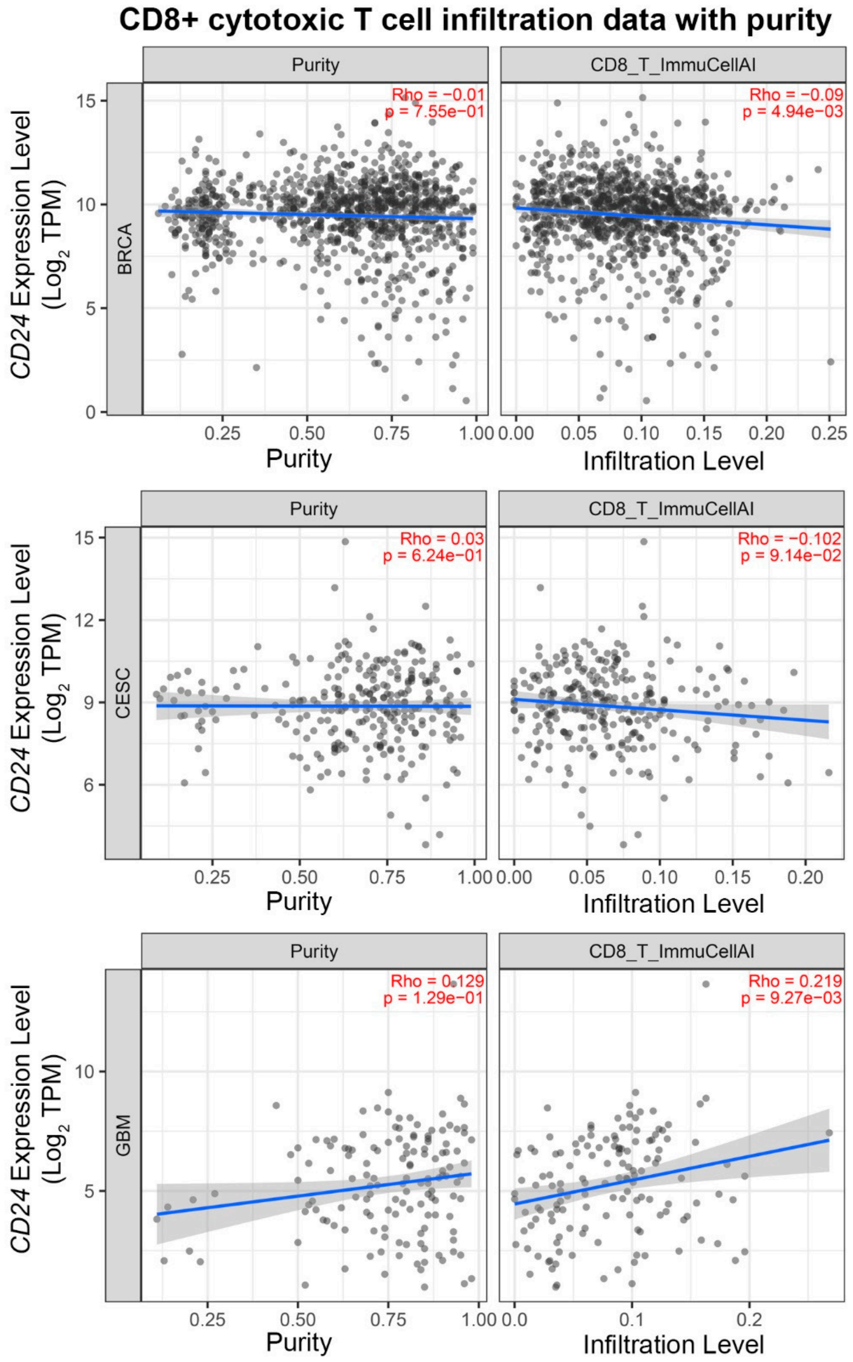

Supplementary Figure S4: CD8+ cytotoxic T cell infiltration level and its association with CD24 expression, adjusted to tumor purity.

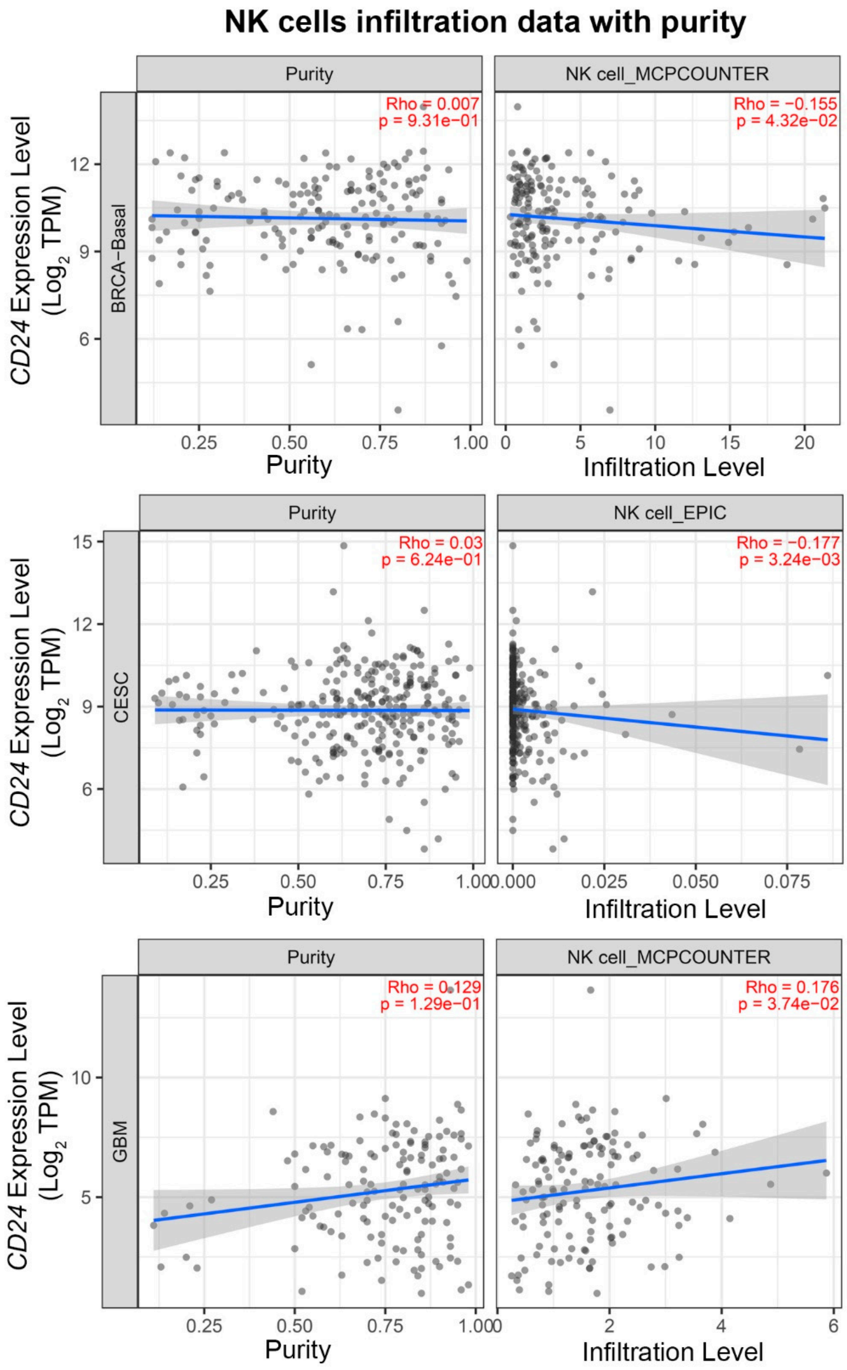

Supplementary Figure S5: NK cell infiltration level and its association with CD24 expression, adjusted to tumor purity
